# *Actinotignum*-associated prosthetic aortic valve endocarditis complicated by postoperative circulatory collapse and dialysis-requiring acute kidney injury: a case report

**DOI:** 10.1186/s12879-026-14146-0

**Published:** 2026-08-03

**Authors:** Norihiro Sakai, Aya Seki, Tomohiro Michino

**Affiliations:** Department of Anesthesiology and Intensive Care Medicine, Daiyukai General Hospital, Sakura 1-9-9, Ichinomiya, Aichi 491-8551 Japan

**Keywords:** *Actinotignum*, Prosthetic valve endocarditis, MALDI-TOF mass spectrometry, Acute kidney injury, Case report

## Abstract

**Background:**

*Actinotignum* species are fastidious genitourinary organisms increasingly recognized in urinary tract and bloodstream infections, but infective endocarditis remains exceptionally uncommon and may be overlooked when a plausible extracardiac infectious focus is present.

**Case presentation:**

A 71-year-old man with recurrent urinary retention requiring clean intermittent self-catheterization presented 7 months after bioprosthetic aortic valve replacement with fever, severe back pain, and dialysis-requiring acute kidney injury. Magnetic resonance imaging showed T12/L1 spondylodiscitis, providing a plausible extracardiac infectious focus. Echocardiography subsequently revealed a large, highly mobile prosthetic-valve vegetation with outflow obstruction. Urgent surgery was undertaken before definitive microbiological identification because of progressive organ dysfunction and concern that further deterioration would reduce operability. Dense periprosthetic induration made redo valve replacement prohibitively hazardous, and extensive debridement with prosthesis retention was used as a nonstandard, survival-prioritizing fallback. The anaerobic bottle from each of two blood culture sets became positive after 92 h, and *Actinotignum* spp. were also recovered from urine. Together with the patient’s urologic risk factors, these findings supported a probable urogenital source. *A. sanguinis* was the highest-ranking matrix-assisted laser desorption/ionization time-of-flight mass spectrometry (MALDI-TOF MS) match for the blood and urinary isolates, but species identity and strain relatedness were not confirmed by molecular or genomic analysis. Empirical ceftriaxone and linezolid were narrowed to intravenous ampicillin after identification and susceptibility review. Later broad-spectrum therapy treated a secondary *Pseudomonas aeruginosa* respiratory infection, and intravenous antibacterial therapy was completed on Day 40. Postoperatively, the pre-existing acute kidney injury progressed to anuria, with recurrent renal replacement therapy circuit clotting and circulatory collapse characterized by predominant low-output physiology. After staged multiorgan support, the patient achieved partial renal recovery with sustained dialysis independence and independent ambulation.

**Conclusions:**

A plausible extracardiac infectious focus should not preclude evaluation for prosthetic valve endocarditis, and urgent source control may need to precede definitive microbiological characterization. Redo valve replacement remains standard; prosthesis-preserving debridement should be regarded only as an individualized, nonstandard fallback when standard surgery becomes prohibitively hazardous. Species-level interpretation of *Actinotignum* identified by MALDI-TOF MS requires caution without confirmatory molecular or genomic analysis.

## Background

Prosthetic valve endocarditis is a severe form of infective endocarditis that requires timely diagnosis, multidisciplinary assessment, and, in selected patients, surgical source control. Recognition may be difficult when clinical manifestations are nonspecific or when a plausible extracardiac infectious focus is identified. These challenges are amplified when the causative organism is fastidious, slow-growing, or not reliably detected or identified by routine microbiological methods. In such circumstances, antimicrobial therapy and indicated surgical source control may need to proceed before complete microbiological characterization is available [1].

*Actinotignum* species are fastidious Gram-positive rods associated predominantly with the genitourinary tract, particularly in older patients with structural or functional urologic abnormalities [2, 3]. Urinary tract and bloodstream infections caused by this genus are increasingly recognized with improved microbiological identification methods [2–4], whereas infective endocarditis remains exceptionally uncommon [5–9] (Table 1). We report a case of *Actinotignum*-associated prosthetic aortic valve endocarditis that illustrates diagnostic uncertainty and individualized source-control decision-making in a critically ill patient.

**Table 1:** Previously reported *Actinobaculum*/*Actinotignum*-associated infective endocarditis cases and the present case

Reference / year	Age / sex	Valve / prosthetic material	Urogenital background / presumed source	Species identification	Molecular confirmation	Local cardiac complication	Shock / AKI or other major systemic complication	Surgical intervention	Antimicrobial treatment	Total antimicrobial duration	Outcome
Hoenigl et al. / 2010 [5]	52 / Male	Prosthetic: Mechanical aortic valve	No underlying urological condition identified	*A. schaalii* via 16 S rRNA gene analysis	16 S rRNA gene analysis (partial sequence, 730 bp)	Filiform vegetation	Middle cerebral artery stroke; fever of unknown origin	None (heart surgeons declined to replace the prosthetic valve)	IV piperacillin-tazobactam → oral amoxicillin-clavulanate	Not reported (Total); Drug-specific: 8 weeks of oral amoxicillin–clavulanate following initial IV therapy	Improved
Diallo et al. / 2018 [6]	63 / Male	Native: Tricuspid and Aortic	BPH; transurethral resection of the prostate 3 weeks before presentation; *A. schaalii* recovered from urine	*A. schaalii* via Vitek MS and 16 S rRNA gene PCR	Broad-range bacterial 16 S rRNA gene PCR performed on fresh valve specimens	Tricuspid vegetation (2 × 2 cm) and grade 3 aortic regurgitation	Acute heart failure	Tricuspid and aortic valve replacement (with bioprosthetic valves)	IV amoxicillin + cefazolin + gentamicin; cefazolin stopped after 5 days	28 days total; gentamicin for 14 days and amoxicillin for 28 days	Complete clinical and microbiological recovery
Loïez et al. / 2018 [7]	89 / Male	Native: Aortic	History of prostatism; no urinary symptoms or documented UTI	*A. schaalii* via MALDI-TOF MS and 16 S rDNA sequencing	16 S rDNA sequencing (419 bp fragment)	Large vegetation (21 mm)	Acute heart failure; postoperative diaphragmatic paralysis requiring ventilatory assistance	Bioprosthetic aortic valve replacement	Amoxicillin/amoxicillin–clavulanate + gentamicin → amoxicillin (12 g/day)	Not reported	Improved; diaphragmatic paralysis gradually improved with physiotherapy
Premkumar et al. / 2022 [8]	86 / Female	Native: Mitral	Previous *E. coli* UTI; *A. schaalii* and *E. coli* recovered from urine	*A. schaalii* recovered from blood and urine; identification method not reported	Not reported	Mitral valve vegetation (2.19 × 0.4 cm)	Peripheral IE stigmata: Janeway lesions, Osler nodes, and splinter hemorrhage	None reported	Ceftriaxone	Not reported	Died from nosocomial pneumonia during treatment
Rallet et al. / 2025 [9]	54 / Male	Prosthetic: Mechanical aortic valve (Bentall graft)	No source identified; urine sterile	*A. schaalii* identified from a prior intraoperative isolate by MALDI-TOF MS and from current blood cultures by Vitek 2 ANC (97% probability)	None reported	Perivalvular abscess fistulizing into the sinus of Valsalva and prosthetic graft; complete Bentall conduit dehiscence with extensive aorto-mitral trigone destruction	Recurrent embolic strokes	Surgical reintervention attempted; reconstruction deemed unfeasible because of complete conduit dehiscence and extensive tissue destruction	Empiric IV amoxicillin + cloxacillin + gentamicin → targeted IV amoxicillin monotherapy (12 g/day)	8 days (reported in the authors’ summary table)	Died after reconstruction was deemed unfeasible
**Present case / 2026** ^b^	71 / Male	Bioprosthetic aortic valve (AVR 7 months earlier)	BPH, recurrent urinary retention, and repeated clean intermittent self-catheterization; probable urogenital source	*Actinotignum* spp. recovered from two separately collected blood culture sets and urine; *A. sanguinis* was the highest-ranking MALDI-TOF MS match for the two blood isolates and one urinary isolate	None; neither 16 S rRNA gene sequencing nor whole-genome sequencing was performed ^a^	Highly mobile prosthetic-valve vegetation (20 × 9 mm) with vegetation-related outflow obstruction; periprosthetic and subvalvular vegetative/granulation tissue; no prosthetic dehiscence	Dialysis-requiring AKI; postoperative circulatory collapse with predominant low-output physiology; recurrent renal replacement therapy circuit clotting	Prosthesis-preserving extensive debridement as a nonstandard intraoperative fallback; SVG–RCA bypass for right coronary ostial injury	IV ceftriaxone plus linezolid → IV ampicillin; later piperacillin–tazobactam, empirical micafungin, and meropenem for secondary *P. aeruginosa* respiratory infection	40 days of continuous IV antibacterial therapy; no oral antimicrobial therapy thereafter ^c^	Improved; partial renal recovery with sustained dialysis independence and independent ambulation; no clinical or microbiological evidence of recurrent IE during two months of follow-up after discharge

Abbreviations: AKI, acute kidney injury; ANC, anaerobe and Corynebacterium identification card; AVR, aortic valve replacement; BPH, benign prostatic hyperplasia; bp, base pairs; IE, infective endocarditis; IV, intravenous; MALDI-TOF MS, matrix-assisted laser desorption/ionization time-of-flight mass spectrometry; MS, mass spectrometry; PCR, polymerase chain reaction; RCA, right coronary artery; rDNA, ribosomal DNA; rRNA, ribosomal RNA; RRT, renal replacement therapy; SVG, saphenous vein graft; UTI, urinary tract infection

^a^ For the present case, species-level molecular or genomic confirmation was not performed. The isolates are therefore reported as *Actinotignum* spp., with *A. sanguinis* described only as the highest-ranking MALDI-TOF MS match

^b^ Among the cases summarized, only the present case combined recovery of *Actinotignum* spp. from both blood and urine with prosthesis-preserving extensive debridement used as a nonstandard intraoperative fallback, postoperative circulatory collapse with predominant low-output physiology, and dialysis-requiring acute kidney injury followed by sustained dialysis independence and independent ambulation

^c^ For the present case, the 40-day duration denotes continuous intravenous antibacterial therapy from Day 1 through Day 40. Later broad-spectrum regimens were administered for a secondary *Pseudomonas aeruginosa* respiratory infection, and no oral antimicrobial therapy was prescribed thereafter

## Case presentation

A 71-year-old man was admitted with fever, severe back pain, and intermittent altered consciousness 7 months after bioprosthetic aortic valve replacement. His medical history included benign prostatic hyperplasia with recurrent urinary retention requiring clean intermittent self-catheterization, a horseshoe kidney, and chronic kidney disease. On admission, he had sepsis with severe acute kidney injury. All subsequent day numbers refer to hospital days, with Day 1 denoting the day of admission. 

 Blood cultures and a urine specimen obtained at urethral catheterization were collected on admission. Given his urologic history and suspected urinary tract infection, intravenous ceftriaxone at 1 g every 24 h was started empirically on Day 1. Severe acute kidney injury with hyperkalemia (serum potassium, 7.1 mmol/L) required urgent hemodialysis. Magnetic resonance imaging (MRI) performed on Day 3 for severe back pain showed pyogenic spondylodiscitis involving the T12/L1 disc and adjacent L1 vertebra, with minimal vertebral destruction and no epidural or paraspinal abscess or neurological deficit. Conservative management with a corset during mobilization was selected. For the suspected spinal infection, intravenous linezolid at 600 mg every 12 h was added; vancomycin was avoided because of the severe renal dysfunction. Fever repeatedly exceeded 38.0 °C, reaching a maximum of 39.0 °C, while marked inflammatory abnormalities and progressive renal dysfunction persisted. 

**Fig. 1:**
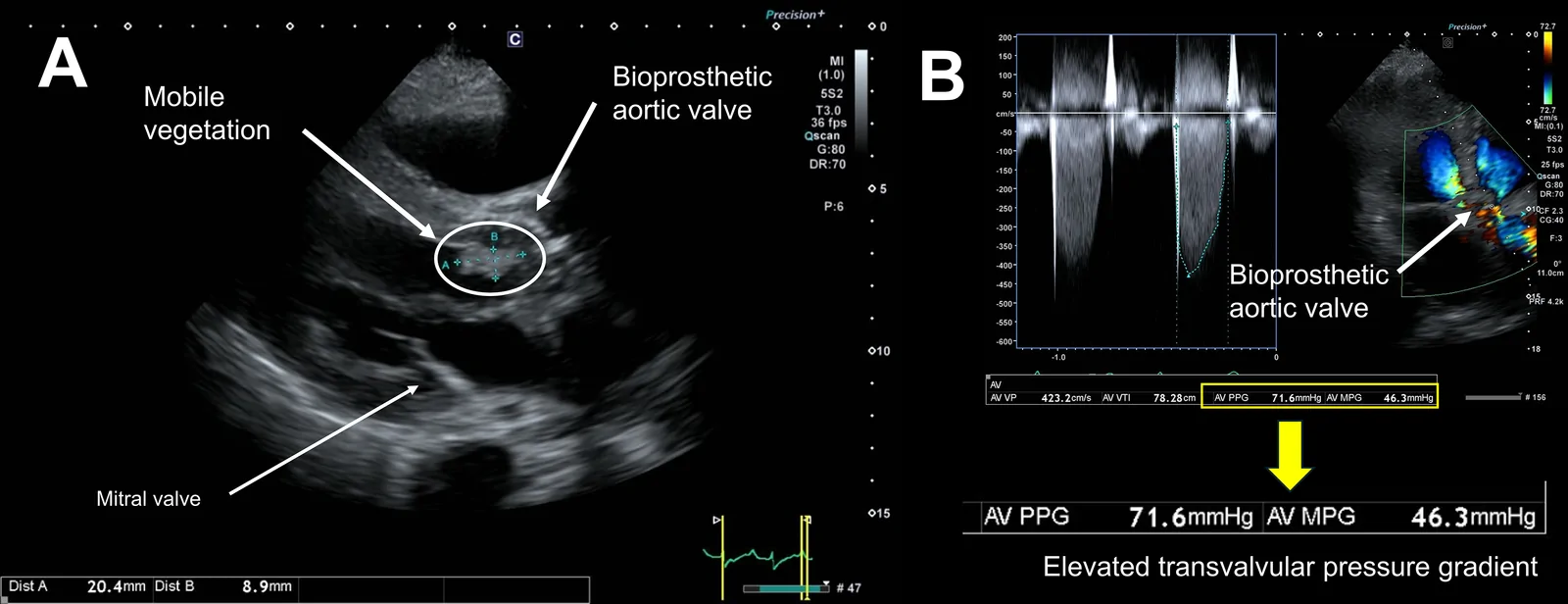
Preoperative transthoracic echocardiography. (**A**) Parasternal long-axis transthoracic echocardiographic image showing a highly mobile echogenic mass measuring 20.4 × 8.9 mm adjacent to the bioprosthetic aortic valve, compatible with vegetation. The annotations identify the bioprosthetic aortic valve, mobile vegetation, and mitral valve. (**B**) Color and spectral Doppler imaging showed an increased peak transprosthetic aortic valve velocity of 4.23 m/s, with peak and mean pressure gradients of 71.6 and 46.3 mmHg, respectively. The annotations identify the bioprosthetic aortic valve and elevated transvalvular pressure gradient. AV, aortic valve; MPG, mean pressure gradient; PPG, peak pressure gradient

**Fig. 2:**
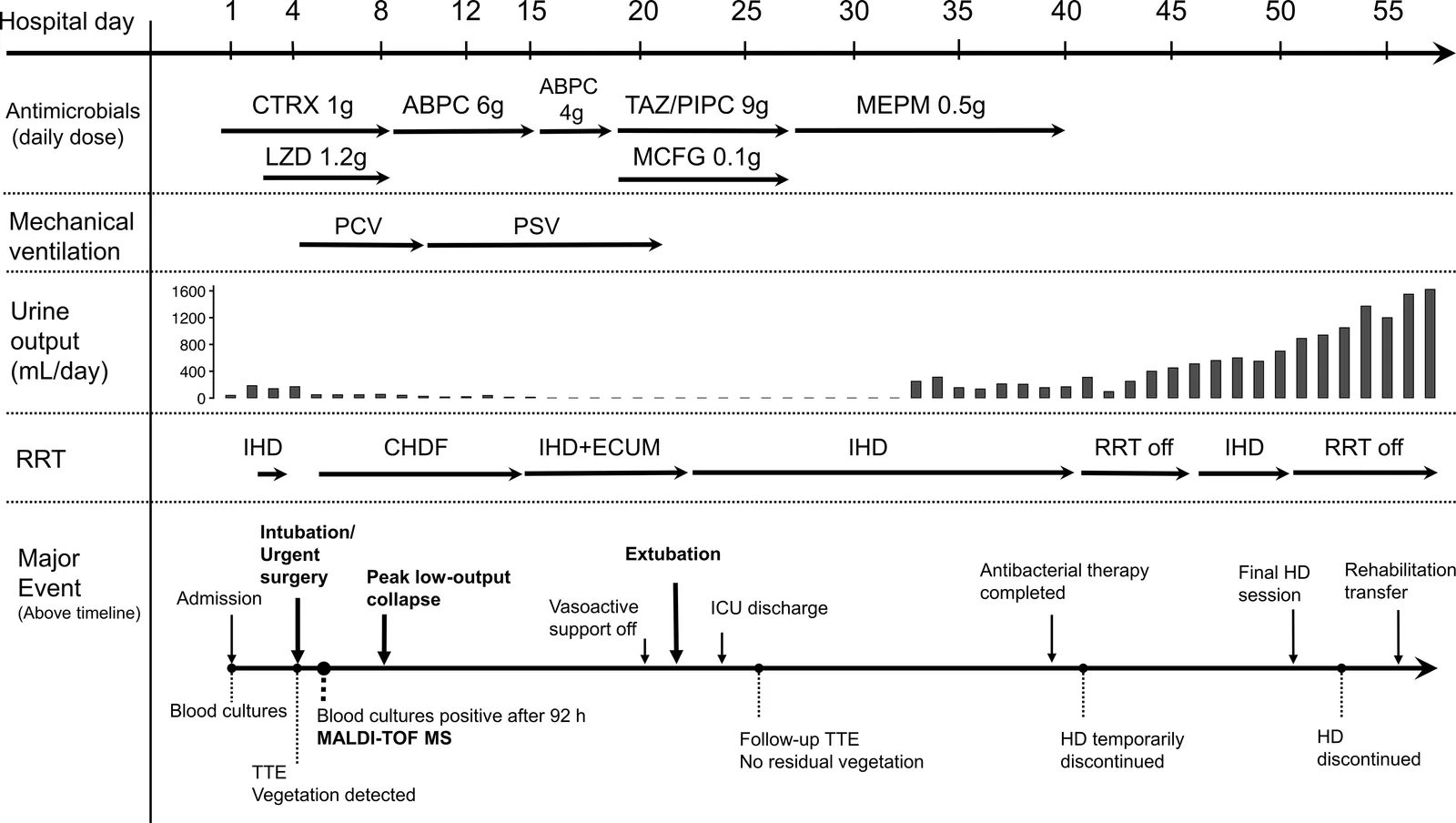
Overall clinical course from admission to transfer to inpatient rehabilitation. Day 1 denotes the day of hospital admission. The figure summarizes antimicrobial therapy, mechanical ventilation, urine output, renal replacement therapy modalities, and major clinical events. Major events include detection of the prosthetic-valve vegetation, urgent surgery, delayed blood-culture positivity after 92 h, MALDI-TOF MS reporting, postoperative circulatory collapse with predominant low-output physiology, ICU discharge, completion of intravenous antibacterial therapy, the final hemodialysis session, discontinuation of dialysis, and transfer to inpatient rehabilitation. ABPC, ampicillin; CHDF, continuous hemodiafiltration; CTRX, ceftriaxone; ECUM, extracorporeal ultrafiltration method; HD, hemodialysis; IHD, intermittent hemodialysis; LZD, linezolid; MALDI-TOF MS, matrix-assisted laser desorption/ionization time-of-flight mass spectrometry; MCFG, micafungin; MEPM, meropenem; PCV, pressure-controlled ventilation; PSV, pressure-support ventilation; RRT, renal replacement therapy; TAZ/PIPC, piperacillin–tazobactam; TTE, transthoracic echocardiography

In retrospect, the spinal findings provided a plausible extracardiac infectious focus but did not fully explain the persistent fever, marked inflammatory abnormalities, and progressive renal dysfunction. Transthoracic echocardiography was subsequently performed and showed a highly mobile vegetation measuring approximately 20 × 9 mm on the bioprosthetic valve, with an elevated transvalvular pressure gradient attributable predominantly to vegetation-related outflow obstruction (Fig. 1). No prosthetic dehiscence or clinically significant aortic regurgitation was observed. Transient hypotension, tachycardia, and brief altered consciousness raised concern for further hemodynamic deterioration and possible embolic complications, although no definite embolic lesion was identified. Given the large mobile vegetation, progressive renal dysfunction, need for source control, and concern that further deterioration would substantially reduce operability, urgent surgery was undertaken without delay before definitive microbiological identification was available (Fig. 2).

Intraoperatively, the bioprosthetic valve showed no leaflet destruction, sewing-ring dehiscence, or suture disruption, but vegetative and granulation tissues were present around the prosthesis and in the subvalvular region. The surrounding aortic tissues were markedly indurated, thickened, and densely adherent. Preoperatively, the patient and family had been informed that redo valve replacement was the preferred standard and would be attempted if feasible, whereas prosthesis-preserving extensive debridement was reserved as a nonstandard fallback if operative risk became prohibitive. Because prosthesis removal required hazardous dissection and appeared likely to necessitate extensive aortic root reconstruction, redo replacement was abandoned. Partial injury to the right coronary ostium during dissection required saphenous vein graft bypass, further increasing procedural risk. A Bentall procedure was therefore not pursued, and the fallback strategy was adopted, with extensive debridement of accessible abnormal tissue while retaining the structurally intact prosthesis. 

Three intraoperative specimens obtained from the periprosthetic, subvalvular, and sternal tissues were negative by smear microscopy and aerobic and anaerobic culture. Ceftriaxone had been administered for approximately 3 days and linezolid for approximately 1.5 days before operative sampling. This preoperative antimicrobial exposure may have reduced the culture yield, although its contribution could not be established. Both anaerobic bottles from the two blood culture sets collected on Day 1 became positive after 92 h (Fig. 2), whereas both aerobic bottles remained negative. Subsequent subculture yielded growth under both 5% CO₂ and anaerobic conditions. Matrix-assisted laser desorption/ionization time-of-flight mass spectrometry (MALDI-TOF MS) yielded *A. sanguinis* as the highest-ranking match for the two blood isolates, with scores of 2.02 and 2.10. The initial routine urine culture showed no growth despite Gram-positive and Gram-negative rods on Gram staining. Subsequent microbiological workup of the same urine specimen identified *Actinotignum* spp. (1+), *Veillonella parvula* (1+), and *Peptoniphilus* sp. (2+). The urinary *Actinotignum* isolate, recovered under anaerobic conditions, yielded *A. sanguinis* as the highest-ranking MALDI-TOF MS match, with a score of 2.31. Because 16 S ribosomal RNA (rRNA) gene sequencing was not performed, the isolates were reported at the genus level as *Actinotignum* spp. The blood and urinary isolates had identical minimum inhibitory concentration (MIC) profiles: ≤0.25 µg/mL for ampicillin, ≤ 1 µg/mL for ceftriaxone, and ≤ 2 µg/mL for linezolid. 

Postoperatively, the pre-existing dialysis-requiring acute kidney injury progressed to anuria, necessitating continuous hemodiafiltration. Recurrent circuit clotting repeatedly interrupted renal replacement therapy and limited sustained fluid removal amid progressive fluid accumulation. 

**Fig. 3:**
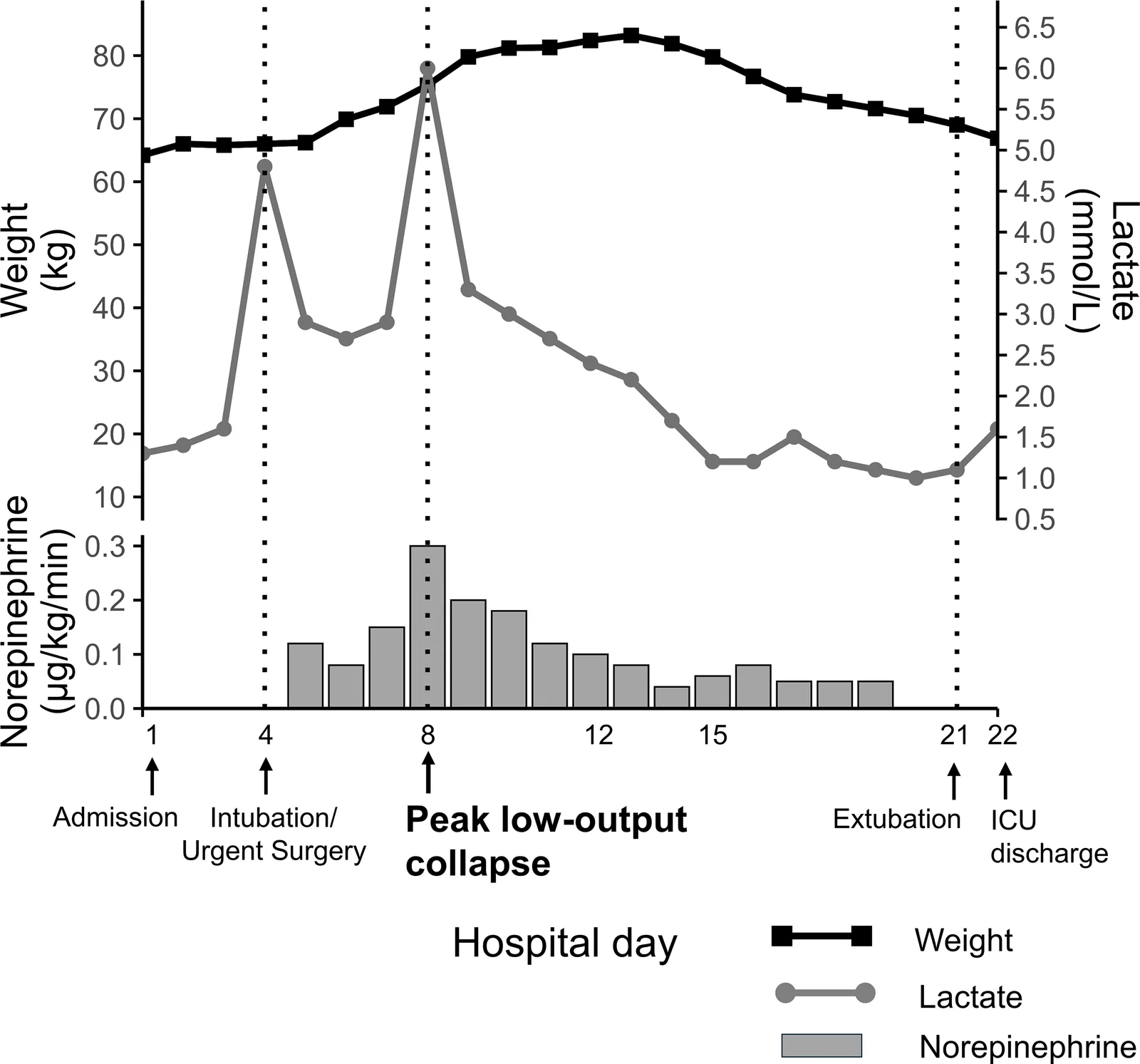
Trends in body weight, blood lactate concentration, and norepinephrine dose during the early ICU course. Day 1 denotes the day of hospital admission. Body weight is shown by the black line with square markers using the left y-axis, and blood lactate concentration is shown by the gray line with circular markers using the right y-axis. Gray bars indicate the maximum norepinephrine dose administered on each hospital day, expressed in µg/kg/min without scaling or transformation. Dotted vertical lines indicate intubation and urgent surgery, peak postoperative low-output circulatory collapse, and extubation. Event annotations additionally identify admission and ICU discharge. ICU, intensive care unit

On Day 8, the patient developed abrupt circulatory collapse, with a blood pressure of 65/30 mmHg, a serum lactate concentration of 6.0 mmol/L, and persistent anuria. FloTrac and PreSep monitoring showed a cardiac output of 2.4 L/min, cardiac index of 1.0 L/min/m², stroke volume of 30 mL, central venous oxygen saturation of 45%, and systemic vascular resistance index of 2450 dyn·s·cm⁻⁵·m². Transthoracic echocardiography demonstrated global biventricular hypokinesis with a left ventricular ejection fraction in the 30% range, without clinically significant prosthetic valve stenosis or regurgitation. Central venous pressure was transiently 35 mmHg immediately after collapse but decreased to 8 mmHg after restoration of arterial pressure. Hemodynamic support required norepinephrine, dobutamine, epinephrine, and vasopressin. These findings indicated predominant low-output physiology, although concomitant vasodilation could not be excluded (Figs. 2 and 3).

Following intensive vasoactive and volume support, hemodynamic stability improved sufficiently to restart continuous hemodiafiltration on Day 8. Ceftriaxone and linezolid were continued during the immediate stabilization period. After *Actinotignum* identification, MIC review, and infectious diseases consultation, treatment was narrowed to intravenous ampicillin on Day 9. Recurrent circuit clotting subsequently decreased, permitting sustained renal replacement therapy and resumption of ultrafiltration on Day 11. The patient was transitioned from continuous hemodiafiltration to intermittent hemodialysis on Day 15 (Figs. 2 and 3). On Day 18, antibacterial therapy was broadened to piperacillin–tazobactam for a secondary *Pseudomonas aeruginosa* respiratory infection, and empirical micafungin was added because fungal coinfection was considered, although no invasive fungal infection was confirmed. With progressive hemodynamic and respiratory improvement, vasoactive support and mechanical ventilation were discontinued, and the patient was transferred from the intensive care unit (ICU) to the ward on Day 22. A repeat respiratory isolate resistant to piperacillin–tazobactam prompted a change to meropenem on Day 28. The antimicrobial sequence and rationale are summarized in Table 2; Fig. 2.

**Table 2:** Antimicrobial treatment course and rationale

Treatment phase	Antimicrobial regimen	Hospital days (duration)	Clinical indication	Microbiological target or basis	Reason for initiation or change
Empirical initial therapy	Ceftriaxone, 1 g IV every 24 h	Day 1–8 (8 days)	Suspected urinary tract infection with sepsis	No organism identified at initiation; blood and urine cultures pending	Started on admission because of the urologic history and suspected urinary tract infection. Discontinued when therapy was narrowed to ampicillin after *Actinotignum* identification, MIC review, and infectious diseases consultation.
Empirical initial therapy	Linezolid, 600 mg IV every 12 h	Day 3–8 (6 days)	Suspected pyogenic spondylodiscitis	Empirical Gram-positive coverage; vancomycin was avoided because of severe renal dysfunction	Added after MRI demonstrated T12/L1 spondylodiscitis. Discontinued when targeted ampicillin therapy was initiated.
Targeted therapy	Ampicillin, 2 g IV every 8 h; reduced to every 12 h from Day 15	Day 9–17 (9 days)	*Actinotignum*-associated prosthetic valve endocarditis with concomitant spondylodiscitis	*Actinotignum spp.*; ampicillin MIC ≤ 0.25 µg/mL	Initiated after *Actinotignum* identification, MIC review, and infectious diseases consultation. Discontinued on Day 17 when antibacterial therapy was broadened on Day 18 for a secondary *Pseudomonas aeruginosa* respiratory infection.
Secondary respiratory infection therapy	Piperacillin-tazobactam, 4.5 g IV every 12 h	Day 18–27 (10 days)	Secondary *Pseudomonas aeruginosa* respiratory infection	*Pseudomonas aeruginosa*	Started for the secondary respiratory infection. Changed to meropenem after a repeat respiratory isolate showed resistance to piperacillin-tazobactam.
Empirical antifungal therapy	Micafungin, 0.1 g IV once daily	Day 18–27 (10 days)	Possible fungal coinfection	Empirical fungal coverage; no invasive fungal infection was confirmed	Added when fungal coinfection was considered and stopped on Day 27. No invasive fungal infection was confirmed.
Secondary respiratory infection therapy	Meropenem, 0.5 g IV every 24 h	Day 28–40 (13 days)	Secondary *Pseudomonas aeruginosa* respiratory infection	Repeat *Pseudomonas aeruginosa* respiratory isolate resistant to piperacillin-tazobactam	Initiated because of resistance to piperacillin-tazobactam and continued through completion of the intravenous antibacterial course on Day 40.

Notes: Intravenous antibacterial therapy was administered continuously from Day 1 through Day 40. Regimen changes after Day 17 were made for a secondary *Pseudomonas aeruginosa* respiratory infection; no microbiological failure of treatment for *Actinotignum*-associated prosthetic valve endocarditis was documented. No oral antimicrobial therapy was prescribed thereafter

MICs for the blood and urinary *Actinotignum* isolates were identical: ampicillin ≤ 0.25 µg/mL, ceftriaxone ≤ 1 µg/mL, and linezolid ≤ 2 µg/mL

Abbreviations: IV, intravenous; MIC, minimum inhibitory concentration; MRI, magnetic resonance imaging

**Fig. 4:**
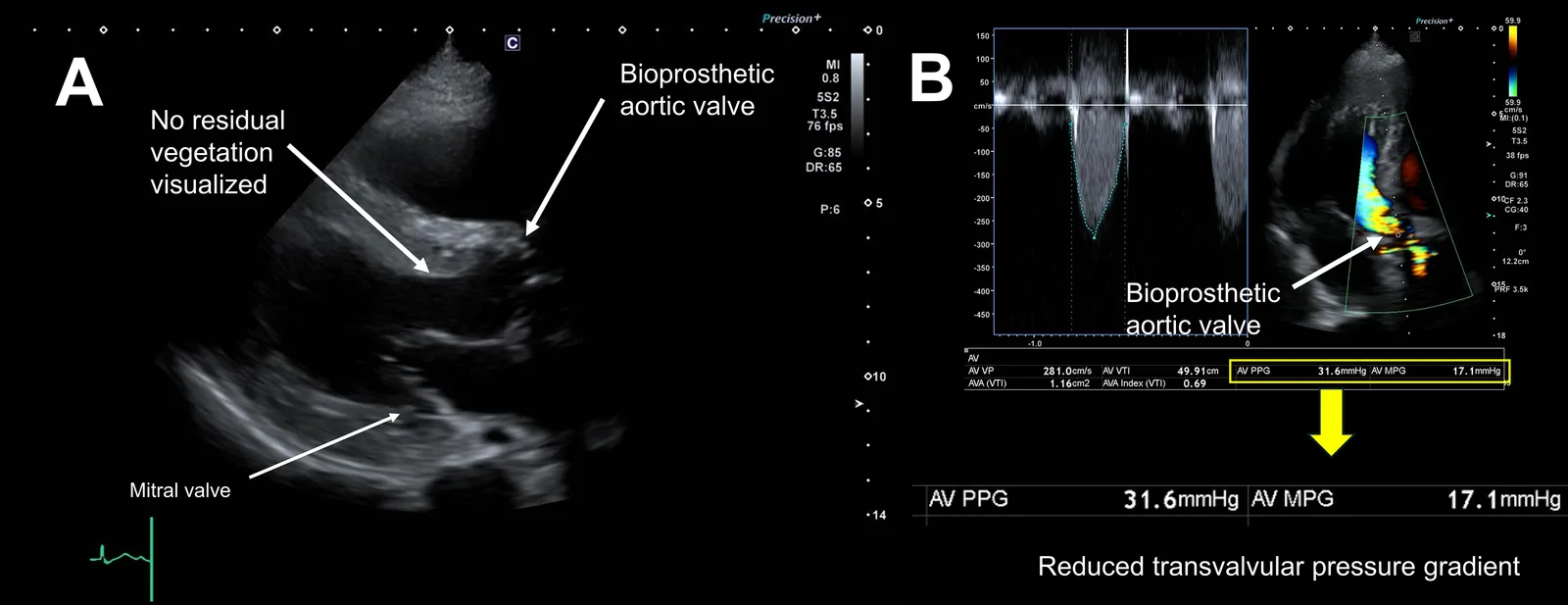
Follow-up transthoracic echocardiography. (**A**) Follow-up transthoracic echocardiographic image showing the retained bioprosthetic aortic valve, with no residual vegetation visualized. The annotations identify the bioprosthetic aortic valve, mitral valve, and region where the vegetation had previously been observed. (**B**) Color and spectral Doppler imaging showed improvement in the peak transprosthetic aortic valve velocity to 2.81 m/s, with peak and mean pressure gradients of 31.6 and 17.1 mmHg, respectively. The annotations identify the bioprosthetic aortic valve and reduced transvalvular pressure gradient. AV, aortic valve; MPG, mean pressure gradient; PPG, peak pressure gradient

Follow-up transthoracic echocardiography on Days 27 and 32 showed no residual vegetation or prosthetic valve dehiscence, preserved prosthetic valve function, improved transvalvular gradients, and recovery of the left ventricular ejection fraction to 51% (Fig. 4). Active gait training began around Day 28. Follow-up MRI on Day 37 showed decreased abnormal signal intensity in the T12/L1 disc without progression of vertebral destruction. Intravenous antibacterial therapy was administered continuously from Day 1 through Day 40, and no oral antimicrobial therapy was prescribed thereafter. The final hemodialysis session was performed on Day 50, and dialysis was discontinued on Day 54 with partial renal recovery (Fig. 2). After acute medical management was completed, the patient was transferred on Day 60 to an inpatient rehabilitation unit within the same institution and discharged home on Day 100 with independent ambulation and sustained dialysis independence. No clinical or microbiological evidence of recurrent infective endocarditis was identified during two months of follow-up after discharge.

### Patient perspective

During the consent interview for publication, the patient and his wife expressed gratitude for his survival and recovery, particularly his return to independent ambulation and sustained dialysis independence. They also wished to proceed with further treatment for recurrent urinary retention and benign prostatic hyperplasia after stabilization, and urological follow-up was arranged accordingly.

## Discussion and conclusion

This case illustrates the diagnostic and therapeutic complexity of *Actinotignum*-associated prosthetic valve endocarditis. Three principal educational points emerge. First, concordant recovery of *Actinotignum* from blood and urine strongly supported its causal role and a probable urogenital source, although species-level identification based on MALDI-TOF MS alone required caution. Second, a plausible extracardiac infectious focus should not preclude evaluation for concomitant prosthetic valve endocarditis, and urgent source control may need to precede definitive microbiological characterization when the window for intervention is narrowing. Third, when redo valve replacement becomes prohibitively hazardous, prosthesis-preserving debridement may be used only as a nonstandard, survival-prioritizing fallback, while postoperative recovery may require serial physiological reassessment and staged multiorgan support.

The few previously reported cases of *Actinotignum*-associated infective endocarditis are summarized in Table 1 [5–9]. Older age, male sex, and urologic disease or instrumentation are also recurring features in the broader literature on *Actinotignum* infection and bacteremia [2, 3]. These observations make a urogenital portal of entry clinically plausible in the present patient, who had recurrent urinary retention and clean intermittent self-catheterization; however, the small and heterogeneous literature precludes firm epidemiological conclusions. The present case adds to the reported spectrum by describing prosthetic aortic valve endocarditis 7 months after bioprosthetic valve replacement, accompanied by spondylodiscitis, progressive renal dysfunction, urgent risk-adapted surgery, and prolonged multiorgan support.

Recovery of *Actinotignum* from both separately collected blood culture sets and urine, together with concordant MALDI-TOF MS results, identical MIC profiles, and substantial urologic risk factors, strongly supported its causal role and a probable urogenital source. However, the polymicrobial urine culture and absence of molecular strain comparison precluded proof of strain identity or the portal of entry.

Species-level assignment remained uncertain. A four-case series from Japan reported concordant identification of *A. schaalii* by MALDI-TOF MS and 16 S rRNA gene sequencing [10], whereas recent genomic analyses have demonstrated cryptic diversity within the genus and limited species-level discrimination by commercial MALDI-TOF MS databases, even at high identification scores [11]. Because no confirmatory molecular or genomic analysis was performed, we report the isolates as *Actinotignum* spp. and retain *A. sanguinis* only as the highest-ranking MALDI-TOF MS match. Delayed positivity of the anaerobic blood culture bottles and the initially negative routine urine culture further illustrate how this fastidious genus may be missed by standard culture workflows [10, 12].

The spinal MRI findings provided a plausible extracardiac explanation for the patient’s fever and severe back pain. However, spondylodiscitis may coexist with infective endocarditis as either a primary infectious focus or a metastatic complication, and the direction of spread may remain uncertain [1]. Current guidelines recommend echocardiographic assessment for infective endocarditis in patients with definite pyogenic spondylodiscitis and positive blood cultures [1]. In a patient with a recently implanted prosthetic valve, such an extracardiac focus should therefore not preclude evaluation for concomitant prosthetic valve endocarditis when systemic inflammation and organ dysfunction remain insufficiently explained.

According to the 2023 Duke–International Society for Cardiovascular Infectious Diseases (Duke-ISCVID) criteria, this case fulfilled the clinical definition of definite infective endocarditis based on one major imaging criterion and three minor criteria. Echocardiographic demonstration of a prosthetic-valve vegetation constituted the major criterion, whereas the prosthetic valve, fever above 38.0 °C, and positive blood cultures not meeting the major microbiological criterion constituted the minor criteria [13]. Under the 2023 Duke-ISCVID criteria, *Actinotignum* is not classified as a typical infective endocarditis pathogen; because it was recovered from only two separately collected blood culture sets, the microbiological evidence fulfilled a minor rather than a major criterion. Pathological criteria were not met because intraoperative cultures were negative and neither histopathological nor molecular examination of operative specimens was performed. Under the 2023 European Society of Cardiology (ESC) criteria, MRI-confirmed spondylodiscitis additionally qualified as a minor criterion for hematogenous osteoarticular septic dissemination [1].

Definitive microbiological identification was still pending when surgery was considered. Although guidelines identify heart failure, uncontrolled infection, and prevention of embolism as the principal indications for surgery and recommend urgent surgery for vegetations ≥ 10 mm when accompanied by other indications [1], the decision in this case was not based on vegetation size alone. The highly mobile 20 × 9-mm prosthetic-valve vegetation with outflow obstruction, progressive renal dysfunction, transient hemodynamic and neurological deterioration, and concern that further decline would reduce operability collectively favored urgent source control. Surgery was therefore undertaken without delay. This course illustrates that, when cardiac anatomy and clinical trajectory indicate a narrowing window for intervention, surgery should not necessarily await definitive microbiological characterization.

The standard surgical principles for active prosthetic valve endocarditis include removal of the infected prosthesis and suture material, thorough debridement of infected and devitalized tissue, and reconstruction according to the extent of structural destruction [1, 14]. In aortic prosthetic valve endocarditis, redo valve replacement and, when necessary, annular or aortic root reconstruction are therefore generally required. Extensive annular or aortic root involvement may substantially increase the complexity of debridement and reconstruction and the risks of coronary injury or conduction disturbance [14].

In this case, redo aortic valve replacement remained the preferred strategy and was attempted. However, the structurally preserved prosthesis was surrounded by densely adherent abnormal tissue, and removal appeared likely to require hazardous dissection and extensive root reconstruction. Right coronary ostial injury requiring saphenous vein graft bypass further increased the procedural risk, prompting adoption of the preoperatively discussed nonstandard fallback of extensive debridement with prosthesis retention.

Follow-up imaging showed preserved prosthetic valve function, and no clinical or microbiological evidence of recurrent endocarditis was identified during two months of follow-up after discharge. This favorable course does not establish prosthesis-preserving debridement as an alternative standard. Prosthetic material remained in an infected field, and the strategy was chosen only because the immediate risk of complete prosthesis removal and root reconstruction was judged to outweigh the uncertain infectious risk of retaining the prosthesis. It should therefore be regarded as an individualized, survival-prioritizing intraoperative fallback in an exceptionally high-risk patient, not as a generally applicable surgical approach.

The postoperative circulatory collapse was best interpreted as predominant low-output physiology rather than a single fixed shock category. Markedly reduced cardiac index and stroke volume, low central venous oxygen saturation, and global biventricular hypokinesis supported a major low-output component, whereas active infection, recent cardiac surgery, and systemic inflammation provided a plausible context for concomitant vasodilation. Because pulmonary artery catheterization was not performed and measurements were obtained during rapidly evolving conditions under ongoing vasoactive and volume therapy, the relative contributions of these components could not be precisely quantified. The transient increase in central venous pressure immediately after collapse, followed by rapid normalization after restoration of arterial pressure, further illustrates why isolated filling-pressure values require temporal and therapeutic context.

The dialysis-requiring acute kidney injury was likewise multifactorial. It was present before surgery and worsened postoperatively amid low-output circulatory failure, systemic inflammation, and fluid accumulation. Recurrent continuous renal replacement therapy circuit clotting intermittently limited sustained renal support and ultrafiltration, complicating volume management, although its contribution to the circulatory collapse cannot be determined. After hemodynamic stabilization, continuous renal replacement therapy and ultrafiltration were progressively resumed, followed by transition to intermittent hemodialysis. Dialysis was discontinued on Day 54, and the patient achieved partial renal recovery with sustained dialysis independence. This course illustrates the value of serial reassessment and staged organ support despite prolonged dialysis dependence.

## Limitations

This case has five principal limitations. First, species-level identification relied on MALDI-TOF MS without confirmatory molecular or genomic analysis. Neither 16 S rRNA gene sequencing nor whole-genome sequencing was performed. Although *A. sanguinis* was the highest-ranking match for the two blood isolates and the urinary isolate, with scores of 2.02, 2.10, and 2.31, respectively, these results could not reliably distinguish it from *A. schaalii* or other cryptic taxa within the genus. Accordingly, *A. sanguinis* could not be definitively confirmed.

Second, all three intraoperative specimens were negative by smear microscopy and aerobic and anaerobic culture, and neither histopathological examination nor molecular testing of the operative specimens was performed. Ceftriaxone had been administered for approximately 3 days and linezolid for approximately 1.5 days before operative sampling. This exposure may have reduced culture yield, but its contribution cannot be determined. In a previously reported case of *Actinotignum* native-valve endocarditis, broad-range bacterial 16 S rRNA gene polymerase chain reaction (PCR) detected *Actinotignum* DNA in excised valves despite negative valve cultures [6]. Molecular testing of operative specimens could therefore have strengthened etiological attribution in the present case, particularly given the preceding antimicrobial exposure and negative tissue cultures.

Third, although the blood and urinary isolates showed concordant MALDI-TOF MS results and identical MIC profiles, genetic relatedness was not examined. The polymicrobial urine culture also precluded exclusive attribution of the urinary microbiological findings to *Actinotignum* or definitive confirmation of the urinary tract as the portal of entry. Whether the spondylodiscitis was the primary infectious focus or a metastatic complication likewise remained unresolved. In addition, because the precise trigger for echocardiography and the contemporaneous diagnostic reasoning were incompletely documented, the extent to which the spinal findings influenced consideration of prosthetic valve endocarditis cannot be determined retrospectively.

Fourth, pulmonary artery catheterization was not performed, and the available hemodynamic measurements were obtained during rapidly evolving circulatory failure under ongoing treatment; therefore, the relative contributions of myocardial dysfunction and vasodilation could not be precisely quantified.

Fifth, the favorable outcome in this single case cannot establish the efficacy, safety, or general applicability of prosthesis-preserving debridement, and the contributions of surgery, antimicrobial therapy, and multiorgan support cannot be separated. The absence of recurrent endocarditis during two months of follow-up after discharge does not exclude later recurrence, and renal recovery remained partial despite sustained dialysis independence.

## Conclusion

Concordant recovery of *Actinotignum* from blood and urine supported its causal role and a probable urogenital source, although species-level identification remained uncertain without molecular or genomic confirmation. In patients with prosthetic valves, a plausible extracardiac infectious focus should not preclude evaluation for endocarditis, and urgent source control may need to precede definitive microbiological characterization. Redo valve replacement remains standard; in this exceptionally high-risk patient, prosthesis-preserving debridement was an individualized, nonstandard, survival-prioritizing fallback rather than a validated alternative. This case underscores the importance of coordinated microbiological, surgical, and critical-care decision-making under substantial uncertainty.

## Data Availability

The data supporting the findings of this case report are included in the article. Additional patient-level clinical data and source records are not publicly available because their disclosure may compromise patient confidentiality. Requests for access to de-identified data may be directed to the corresponding author and will be considered in accordance with institutional policies and applicable ethical and legal requirements.

## Declarations

### Use of artificial intelligence-assisted tools

Artificial intelligence-assisted tools, including ChatGPT, were used for language editing, manuscript organization, and refinement of English expressions. No artificial intelligence-assisted tool was used to generate or modify clinical data, microbiological data, references, figures, or clinical images. The authors reviewed and verified all content, clinical interpretations, and references, and take full responsibility for the final manuscript.

## Abbreviations

Duke-ISCVID: Duke–international society for cardiovascular infectious diseases
ESC: European society of cardiology
ICU: Intensive care unit
MALDI-TOF MS: Matrix-assisted laser desorption/ionization time-of-flight mass spectrometry
MIC: Minimum inhibitory concentration
MRI: Magnetic resonance imaging
PCR: Polymerase chain reaction
rRNA: ribosomal RNA
